# Supplementary material for: The interactome of intact mitochondria by cross-linking mass spectrometry provides evidence for coexisting respiratory supercomplexes
Source: Mol Cell Proteomics. 2017 Dec 8;17(2):216–32. doi: 10.1074/mcp.RA117.000470 (PMC5795388; doi:10.1074/mcp.RA117.000470)
Supplement: Supplemental Data [file supp_RA117.000470_133922_0_supp_23978_fzffwf.zip › spectra_annotation/mito_spectra_annotation/298-1-4-1-4-1.pdf]

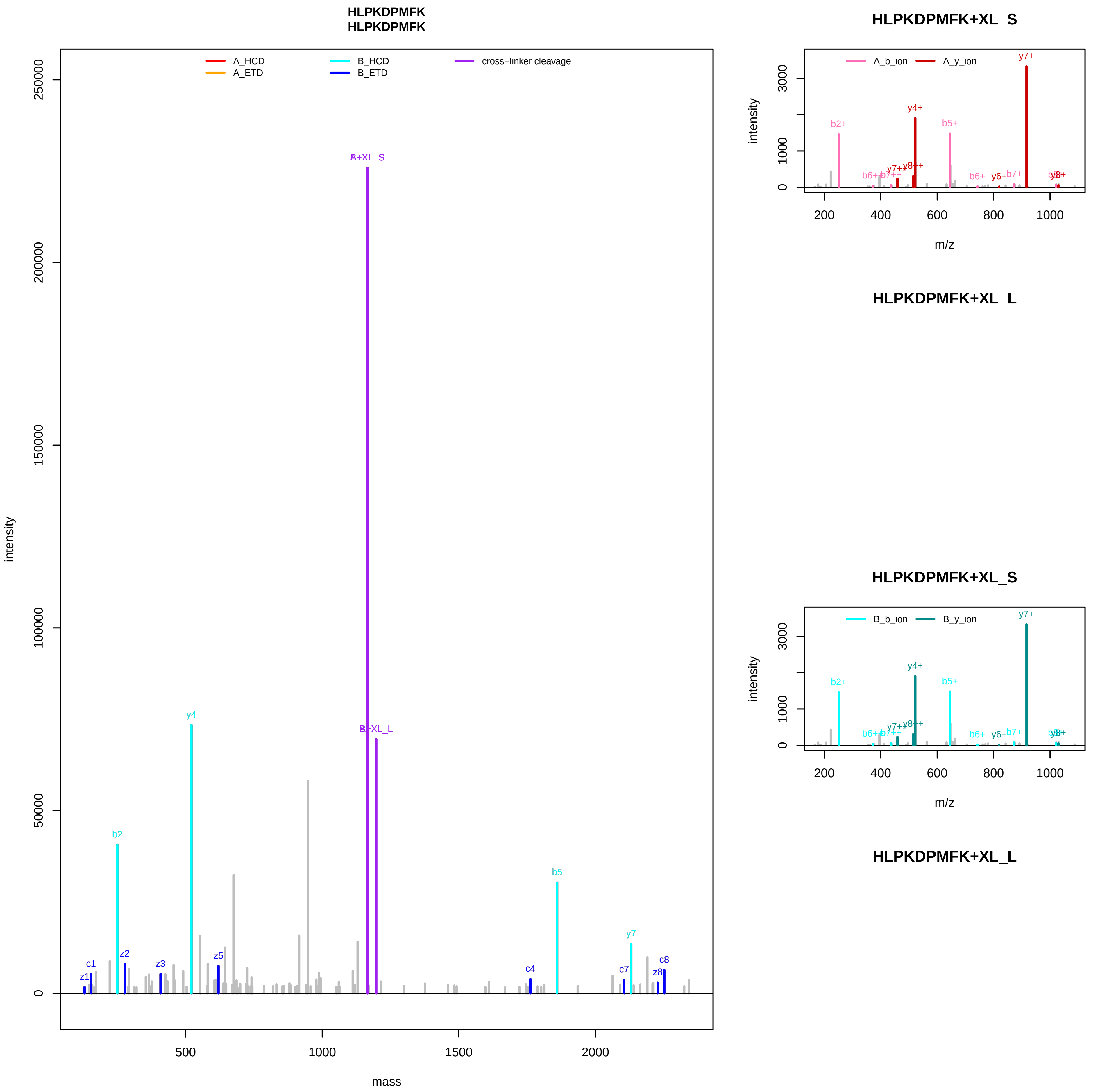
HLPKDPMFK
HLPKDPMFK
A_HCD
A_ETD
B_HCD
B_ETD
cross−linker cleavage
intensity
mass
0
50000
100000
150000
200000
250000
500
1000
1500
2000
B+XL_S
B+XL_L
b2
y4
b5
y7
c1
z1
z2
z3
z5
c4
c7
z8
c8
HLPKDPMFK+XL_S
A_b_ion
A_y_ion
y7+
y4+
b2+
b5+
y7++
y8++
b6++
b7++
b6+
y6+
b7+
y8+
intensity
m/z
0
1000
3000
200
400
600
800
1000
HLPKDPMFK+XL_L
HLPKDPMFK+XL_S
B_b_ion
B_y_ion
y7+
y4+
b2+
b5+
y7++
y8++
b6++
b7++
b6+
y6+
b7+
y8+
intensity
m/z
0
1000
3000
200
400
600
800
1000
HLPKDPMFK+XL_L
